# Role of Disulfide Cross-Linking of Mutant SOD1 in the Formation of Inclusion-Body-Like Structures

**DOI:** 10.1371/journal.pone.0047838

**Published:** 2012-10-31

**Authors:** Brittany L. T. Roberts, Kinaree Patel, Hilda H. Brown, David R. Borchelt

**Affiliations:** Department of Neuroscience, Center for Translational Research in Neurodegenerative Disease, SantaFe HealthCare Alzheimer's Disease Research Center, McKnight Brain Institute, University of Florida, Gainesville, Florida, United States of America; National Institute of Health, United States of America

## Abstract

**Background:**

Pathologic aggregates of superoxide dismutase 1 (SOD1) harboring mutations linked to familial amyotrophic lateral sclerosis (fALS) have been shown to contain aberrant intermolecular disulfide cross-links. In prior studies, we observed that intermolecular bonding was not necessary in the formation of detergent- insoluble SOD1 complexes by mutant SOD1, but we were unable to assess whether this type of bonding may be important for pathologic inclusion formation. In the present study, we visually assess the formation of large inclusions by fusing mutant SOD1 to yellow fluorescent protein (YFP).

**Methodology/Principal Findings:**

Experimental constructs possessing mutations at all cysteine residues in SOD1 (sites 6, 57, 111, and 146 to F,S,Y,R or G,S,Y,R, respectively) were shown to maintain a high propensity of inclusion formation despite the inability to form disulfide cross-links. Interestingly, although aggregates form when all cysteines were mutated, double mutants of the ALS mutation C6G with an experimental mutation C111S exhibited low aggregation propensity.

**Conclusions/Significance:**

Overall, this study is an extension of previous work demonstrating that cysteine residues in mutant SOD1 play a role in modulating aggregation and that intermolecular disulfide bonds are not required to produce large intracellular inclusion-like structures.

## Introduction

Amyotrophic lateral sclerosis (ALS) is a progressive neurodegenerative disease characterized by the loss of upper and lower motor neurons. The disease typically presents with unknown etiology (sporadic ALS), but about 20% of ALS cases are caused by the inheritance of genetic mutations. In recent years, mutations in a number of different genes have been implicated as causative for ALS or motor neuron disease, including the genes encoding AR (androgen receptor), TDP-43 (TAR DNA binding protein), FUS (a component of a fusion protein in malignant liposarcoma), OPTN (optineurin), ANG (angiogenin), ALS2 (alsin – a G-protein exchange factor), FIG4 (FIG4 homolog - phosphatase), SETX (senetaxin – RNA helicase), UBQLN2 (ubiqulin 2 – ubiquitin binding protein), and VCP (Valosin-containing protein) {http://alsod.iop.kcl.ac.uk/default.aspx}. One of the first true ALS genes to be identified was Cu-Zn superoxide dismutase (SOD1) [1], which predominantly shows a dominant inheritance pattern. To date, more than 140 mutations in SOD1 have been identified in cases of fALS {http://alsod.iop.kcl.ac.uk/default.aspx}. The majority of these fALS-linked SOD1 mutations are point mutations. A subset of fALS mutations cause shifts in the reading frame or introduce early termination codons, resulting in the production of C-terminally truncated proteins.

The normal function of SOD1 is to metabolize oxygen radicals that are produced by cellular metabolism. The active enzyme is a homodimer of two 153 amino acid subunits with each subunit containing eight β-strands, an active site that binds copper, a binding site for zinc, an electrostatic loop that funnels in the substrate, and an intramolecular disulfide bond between cysteines 57 and 146 [2], [3]. The effects of fALS mutations on the normal enzyme activity vary considerably [4]–[6]. In cell culture and *in vitro* models, enzyme activity ranges from undetectable to near normal [4], [7]–[10]. Although some mutants are extremely unstable [11], [12] and some mutants are inactive [8], [13], others retain high levels of activity [4], [10], [14]–[16]. Thus, it is generally accepted that mutations in SOD1 do not cause ALS as a result of lost function. Notably, the targeted deletion of SOD1 in mice does not induce ALS-like symptoms [17]. Overall, the preponderance of evidence indicates that fALS-associated mutations in SOD1 cause disease as a consequence of an acquired toxic property.

Multiple studies have demonstrated that one consequence to fALS mutations to SOD1 is that the protein becomes much more prone to aggregate [18]–[22]. Indeed, we have yet to identify an fALS-associated mutant that does not show heightened propensity to form aggregates that are insoluble in nonionic detergent [22]. Studies from several groups have demonstrated that these detergent-insoluble aggregates of SOD1 protein contain molecules that are cross-linked by intermolecular disulfide bonds [23]–[25]. Within each SOD1 subunit, there are four cysteine residues at amino acids 6, 57, 111, and 146 that could potentially form intermolecular disulfide bonds [23], [26], but other evidence suggests that cysteines 6 and 111 may be primarily involved in oxidation-mediated aggregation [27]. However, in prior studies involving culture cell models of aggregation, we have found that disulfide cross-linking is not a critical event in aggregation as artificial mutants that lack cysteine can still form detergent insoluble structures [28].

One of the shortcomings of our prior work is that we relied heavily on a biochemical assay for aggregation in which cell lysates were subjected to extraction in nonionic detergents and centrifugation [28]. This method, although useful, does not provide information on the morphology of the aggregates or how loss of disulfide crosslinking might affect the generation of higher order structures, such as intracellular inclusions. In our hands, with our antibody reagents, visualization of inclusions in cells has proven difficult due to poor accessibility of antibody epitopes [29], [30]. To overcome this problem we adopted a strategy used by others in which SOD1 cDNA was fused in-frame with fluorescent proteins [31]–[37]. In our studies, we used yellow-fluorescent protein (YFP) as a C-terminal fusion protein and we were able to easily visualize the formation of fluorescent inclusions in cells expressing fusions of mutant SOD1 with YFP [30], [33], [38]. In the present study, we have applied this approach to the study of a series of experimental SOD1 mutants in which cysteine residues have been systematically mutated. Our findings confirm and extend our previous work in demonstrating that disulfide cross-linking is not required for the aggregation of mutant SOD1 and that mutant SOD1 lacking cysteine residues can form large intracellular inclusions.

## Methods

### Generation of mutant SOD plasmids

HSOD1 cDNA's containing the mutations C111Y, C111S, C6G/C111Y, C6G/C111S, C6G/C57S/C111Y/C146R (GSYR), and C6F/C57S/C111Y/C146R (FSYR), previously described by Karch et al [28] were subcloned into modified pEF-BOS vector containing YFP cDNA [30] using the In-Fusion Cloning kit (Clontech, Mountain View, CA; Cat. No. 639617). Each mutant was amplified by standard PCR strategies. The PCR product was then analyzed on a 1% agarose gel in order to confirm a single PCR product. Next, an infusion cloning procedure was used to insert the DNA into pEF-BOS vector that already contained the YFP cDNA. These plasmids were transformed into NEB-10β competent cells (New England Biolabs) following standard protocols. Large scale preparations of plasmid DNA for transfection were prepared by CsCl gradient purification. All plasmids were verified by DNA sequence analysis.

### Transient Transfections

Expression plasmid DNA was transiently transfected into Chinese hamster ovary (CHO) cells. The day before the transfection, the CHO cells were split into 60-mm poly-D-lysine-coated dishes (1 plate for each DNA construct). Upon reaching 95% confluency, cells were transfected with Lipofectamine™ 2000 (Invitrogen). The cells were then incubated at 37°C in a CO_2_ incubator for 24 hours. Representative pictures were then taken using an AMG EVOS_fl_ digital inverted microscope for fluorescence and transmitted light applications. Pictures of both typical cells and cells with aggregates were taken at both 20× and 40× magnification at both 24 and 48 hours. The transient transfections were repeated two more times for each construct with representative pictures taken of random fields. The images from multiple transfections were analyzed with cells showing YFP fluorescence, fluorescent inclusions, and fluorescent cells showing condensed morphology counted in a blinded fashion.

## Results

**Figure 1:**
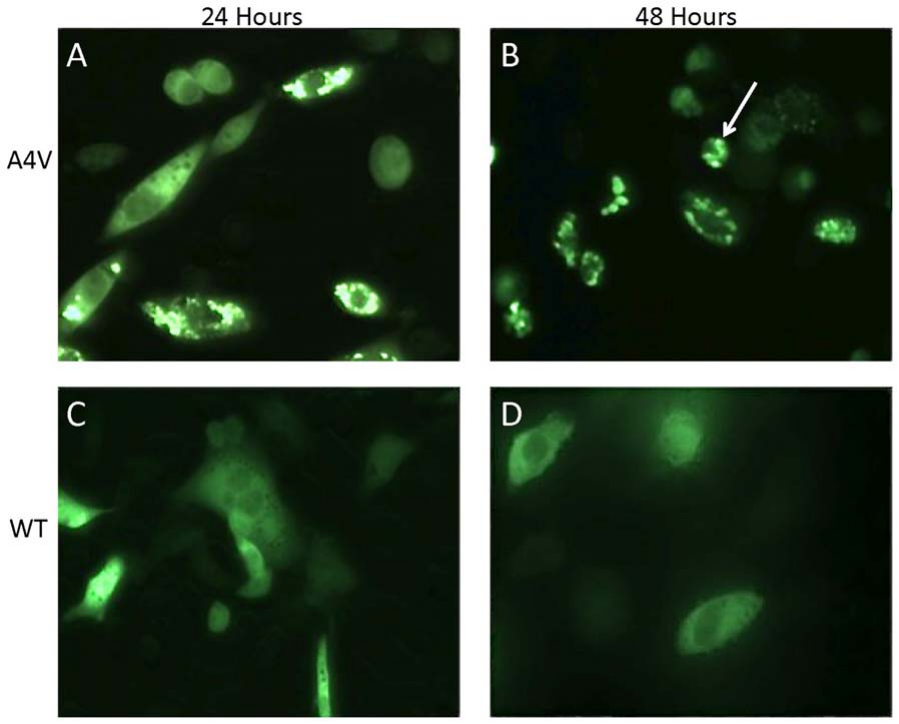
Mutant SOD1:YFP fusion proteins form obvious intracellular inclusions. As described in Methods, CHO cells were transiently transfected with expression plasmids for WT or A4V SOD1 fused to YFP. After 24 or 48 hours the cells were fixed with 4% paraformaldehyde and then images were captured to assess the frequency of inclusion formation. Cells expressing the A4V SOD:YFP fusion protein contain inclusion-like structures at a high frequency.

This study utilized expression of SOD1:YFP fusion proteins as a means to examine the role of disulfide cross-linking in the formation in intracellular inclusions. The ability of various SOD1 mutants fused to YFP to form inclusions was compared to a set of controls that included the SOD1-A4V mutant (Fig. 1A and B, high propensity to form inclusions) and WT SOD1 (Fig. 1C and D, no inclusion formation). In previous work, we have established that YFP alone shows only a diffuse distribution similar to the SOD1-WT:YFP fusion proteins [30], [38]. Three consecutive transient transfections were done for each construct and representative images were captured at both 24 and 48 hours for quantification of aggregate formation (Table 1). Throughout the study, we noted that a percentage of cells expressing the YFP fusion protein adopted a very condensed abnormal morphology (see Figure 1B, arrow). These condensed cells were generally round, much smaller in size, and often were only weakly attached to the plate. Cells exhibiting this morphology were also quantified.

**Table 1:** Aggregation of SOD1 mutants in transfected cells.

SOD1 Construct	Production of inclusions in 24 h	# of cells with YFP counted	# of cells with inclusions	# of condensed cells
WT	−	128	0 (0%)	9 (7%)
A4V	+	314	83 (27%)	36 (11%)
C111Y	+	850	252 (29%)	68 (8%)
C111S	−	747	0 (0%)	142 (19%)
C6G/C111Y	+	632	43 (6%)	50 (8%)
C6G/C111S	−	898	0 (0%)	106 (12%)
FSYR	+	229	117 (51%)	13 (6%)
GSYR	+	257	36 (14%)	21 (8%)

	Production of inclusions in 48 h	# of cells with YFP counted	# of cells with inclusions	# of condensed cells
WT	−	83	0 (0%)	43 (52%)
A4V	+	33	23 (70%)	25 (76%)
C111Y	+	163	107 (67%)	82 (50%)
C111S	−	79	0 (0%)	39 (49%)
C6G/C111Y	+	55	14 (25%)	27 (49%)
C6G/C111S	−	118	0 (0%)	47 (40%)
FSYR	+	50	29 (58%)	40 (80%)
GSYR	+	29	13 (45%)	22 (78%)

**Figure 2:**
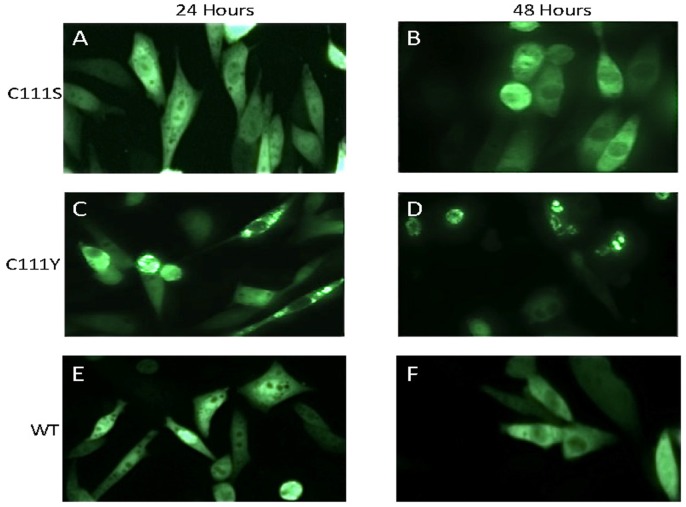
Mutation of cysteine 111 to serine or tyrosine differentially affects inclusion formation. CHO cells were transiently transfected with expression plasmids for each mutant and imaged 24 or 48 hours later as described in Figure 1. Mutation of cysteine 111 to serine does not induce inclusion formation whereas the disease associated mutation of cysteine to tyrosine produces inclusion-like structures at a high frequency. For comparison, cells expressing WT SOD1:YFP fusion proteins are also shown.

Based on prior study [28], [39], we were particularly interested in the role of the cysteine residue located at position 111. Previous work determined that mutation of cysteine 111 to serine does not induce the misfolding of SOD1 to form detergent-insoluble aggregates [28], [40]. As expected, in cells expressing a C111S variant of human SOD1 fused to YFP, we observed no obvious inclusion formation in cells transiently transfected for 24 or 48 hours (Fig. 2A and B) (Table 1). By contrast, cells expressing the fALS-associated mutation C111Y SOD1 fused to YFP we observed obvious inclusion formation by 24 hours (Fig. 2C) with persistent inclusion formation out to 48 hours (Fig. 2D) (Table 1). Cells expressing WT SOD1:YFP fusion protein did not contain inclusions-like structures at either time point (Fig. 2E and F). This outcome was consistent with our previous study in which the C111Y mutant was found to produce detergent-insoluble aggregates [28].

**Figure 3:**
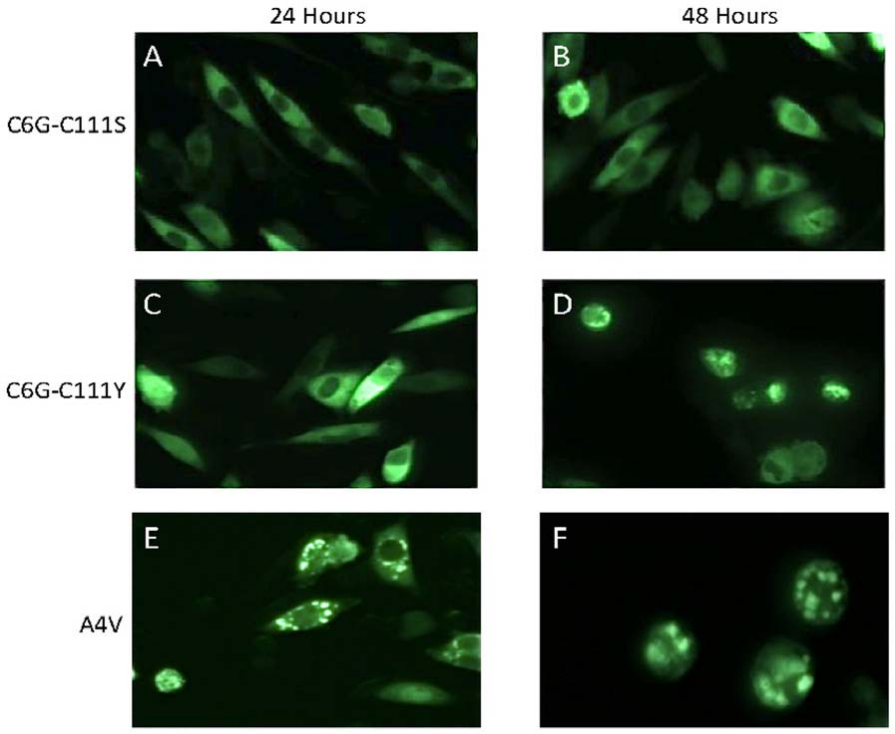
Simultaneous mutation of cysteines 6 and 111. CHO cells were transiently transfected with expression plasmids for each mutant and imaged 24 or 48 hours later as described in Figure 1. For the C6G-C111S and C6G-C111Y mutants (Panels A–D), the rate of inclusion formation was slower than that of the positive control mutant A4V (Panels E and F). At 48 hours, the frequency of inclusion-like structures in cells expressing the double mutant C6G-C111S remained low compared to C6G-C111Y (see Table 1).

The cysteine at position 6 of SOD1 has also been implicated in the formation of disulfide linked aggregates [27], [40]. In previous study, we found that mutation of cysteine 6 to the fALS mutation glycine produced a protein that is prone to form detergent-insoluble aggregates [28]. However, introduction of a second mutation at cysteine 111 to serine suppressed aggregation [28]. Here we examined the same construct as a fusion to YFP along with a second construct combining the C6G mutation with C111Y. At 24 hours, we observed no obvious inclusions in cells expressing SOD1 C6G-C111S:YFP and a relatively low percentage of cells with inclusions by expression of SOD1 C6G-C111Y fusion proteins (Fig. 3A and C) (Table 1). By comparison, cells expressing SOD1 A4V:YFP fusion protein showed obvious inclusions at 24 hours (Fig. 3E). By 48 hours, cells expressing SOD1 C6G-C111S:YFP fusion protein still lacked obvious inclusion-like structures, but there were cells in which the fluorescence appeared to be less than completely uniform (Fig. 3B). At the same time point, cells expressing SOD1 C6G-C111Y:YFP fusion protein showed obvious inclusion-like structures (Fig. 3D); comparable in appearance to cells expressing SOD1 A4V:YFP fusion protein (Fig. 3F). Overall the relative frequency of inclusion formation in cells expressing SOD1- C6G-C111S:YFP was very low (Table 1).

**Figure 4:**
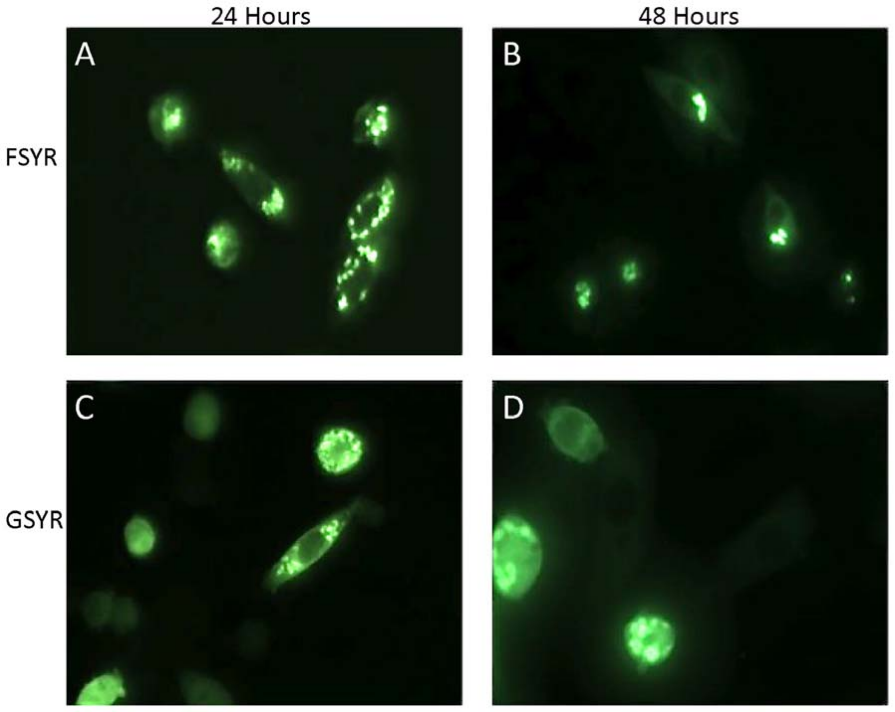
Simultaneous mutation of all four cysteines in SOD1. CHO cells were transiently transfected with expression plasmids for each mutant and imaged 24 or 48 hours later as described in Figure 1. Mutants lacking cysteine formed obvious inclusion-like structures. FSYR; C6F/C57S/C111Y/C146R. GSYR; C6G/C57S/C111Y/C146R.

In prior work, we had generated two experimental constructs in which all four cysteine residues (C6G or F, C57S, C111Y, and C146R) were mutated [28]. Of the 4 positions mutated, only the C57S mutation has not been associated with fALS. We created fusions of the cDNA for these mutants with YFP and expressed them for 24 or 48 hours by transient transfection. The SOD1-FSYR:YFP construct showed a high propensity to form inclusions at 24 hours (Fig. 4A) with persistent inclusion formation out to 48 hours (Fig. 4B). At 24 hours about 50% of the fluorescent cells contained inclusions (Table 1). The GSYR construct, in which cysteine 6 was mutated to glycine, also produced cells with fluorescent inclusions by 24 hours, but the frequency was less than either the FSYR:YFP fusion protein or the A4V:YFP fusion protein (Fig. 4C) (Table 1). By 48 hours, cells expressing SOD1-GSYR:YFP frequently contained inclusions (Fig. 4D). These experimental constructs indicate that the presence of cysteine residues is not essential in the formation of large intracellular inclusions by mutant SOD1.

## Discussion

Our study examined the relationship between intermolecular disulfide bonds and the development of large visible inclusions by mutant superoxide dismutase 1 (SOD1) fused to a YFP reporter protein. Our findings confirm and extend our previous work in which we demonstrated that mutant SOD1 lacking all four cysteine residues can form detergent-insoluble complexes; mutants C6G/C57S/C111Y/C146R (GSYR) and C6F/C57S/C111Y/C146R (FSYR) [28]. However, we had not been able to determine the physical morphology of these aggregates by standard immunostaining approaches. Our new findings demonstrate that large inclusion structures of mutant SOD1 can form in the absence of intermolecular disulfide bonding.

Previous studies have shown that the cysteine residue at position 111 in SOD1 plays an important role in the formation of aggregates [28], [40]. Mutation of C111 to serine has a strong inhibitory influence on the formation of detergent insoluble protein aggregates [28], [39], [40]. However, when C111 is mutated to tyrosine, the mutant SOD1 formed detergent insoluble aggregates [28]. Our findings here confirm this result as we observe that SOD1-C111S fused to YFP does not form obvious inclusions whereas inclusions are readily observed in cells expressing SOD1-C111Y fused to YFP. Indeed, the C111Y variant showed inclusion formation (29% of YFP positive cells) similar to that of the A4V positive control at both 24 and 48 hours (Table 1). In comparison, no inclusions were observed in cells expressing the C111S variant. Thus we confirm that mutation of cysteine 111 to tyrosine produces a protein that can form large intracellular aggregates, indicating that disulfide crosslinking through cysteine 111 is not crucial to aggregate formation.

In a previous study, we had noted that experimental combinations of mutations in which C6G and C111S were combined or when C6G and C111Y were combined, the resultant double mutants produced very low amounts of detergent-insoluble aggregates [28]. In the present study, we find similar results in that C6G-C111S fusion protein showed no inclusion formation at 24 or 48 hours, similar to the WT and C111S constructs. Cells expressing the C6G-C111Y fusion protein showed low levels of inclusion formation at 24 hours (6% of YFP expressing cells) but inclusions became more obvious by 48 hours. Although these findings are consistent with the notion that cysteines 6 and 111 are in some manner involved in the assembly of mutant SOD1 into large multimeric structures, their role is difficult to define because mutants lacking all 4 cysteines readily form inclusion-like structures. Indeed, the SOD1 FSYR:YFP fusion construct is similar to WT SOD1 in terms of aggregation propensity and morphology. Thus, overall, these studies demonstrate that although there are some potentially interesting modulatory roles for cysteines at position 6 and 111 in SOD1 aggregation, mutant SOD1 lacking all 4 cysteines can form large inclusion structures. Thus, intermolecular disulfide crosslinking is not required to produce large assemblies of mutant SOD1.

Throughout this study, we noted that by 24 hours between 5 and 20% of cells expressing SOD1:YFP fusion protein adopted a very condensed abnormal morphology (see Figure 1B, arrow)(see Table 1). By 48 hours, the percentage of cells expressing the YFP fusion protein that showed a condensed morphology was as high as 50%. Similar condensed cells have been noted in prior studies of CHO cells expressing mutant SOD1:YFP fusion proteins [30]. In this prior work, we noted that these cells do not display markers of dead or dying cells [30] and the basis for the morphological change is presently unknown. Notably, there was no obvious correlation between expression of mutant SOD1 and the frequency of condensed cells at 24 hours. Thus, we cannot attribute this change in morphology to a consequence of inclusion formation.

In past efforts to determine whether ALS associated mutations in SOD1 cause the protein to aggregate, we have relied on biochemical assays of detergent insolubility [12], [20]–[22], [25], [28], [38], [39], [41]–[44]. In these biochemical tests, we relied on sonication as a method of cell lysis in order to shear nucleic acid to a size that did not interfere with protein sedimentation by ultracentrifugation. Without some method to shear nucleic acid, the viscosity of the cell lysate can greatly interfere with velocity sedimentation. One issue with this approach is that sonication has the potential to cause protein aggregation if the lysate is overheated during the process. In our past experience in using sonication to lyse cells, WT SOD1 was not induced to aggregate whereas the mutants were all variably prone to aggregate [22]. However, the potential remained that mutant SOD1 was somehow primed to aggregate with this property becoming evident upon sonication. Recently, we have re-examined the aggregation of mutant SOD1 by fusing the mutant protein to YFP [30]. Five different ALS associated mutants were all shown to spontaneously adopt an inclusion like morphology and these mutants all showed increased accumulation of detergent insoluble SOD1:YFP fusion protein [30]. Notably, several studies of cells expressing fusion proteins of mutant SOD1 and fluorescent reporter proteins have demonstrated that mutant SOD1 is more prone to form aggregates [31]–[37], [45]. Our present study provides additional corroborative data in which the behavior of fluorescently tagged SOD1 variants essentially replicates previous findings based on sonication lysis, detergent extraction, and sedimentation. Thus, it is difficult to argue against the notion that one consequence of mutations in SOD1 is to increase propensity to misfold and aggregate. The role of mutant SOD1 aggregation plays in toxicity remains an unanswered question, but the use of fluorescently tagged proteins offers an additional tool in addressing this question.

### Conclusions

Through visual detection of intracellular inclusions and quantitative analysis, we were able to confirm past research that had concluded that intermolecular disulfide bonds are of limited importance in formation of mutant SOD1 aggregates. A caveat to prior work was that it relied completely on assessing detergent insolubility and at the time we were not able to ascertain whether the various manipulations of the cysteine residues may have altered the formation of inclusion body aggregates by immunocytochemistry. Our present study demonstrates that mutant SOD1 lacking all four cysteine residues can form large inclusion body aggregates, indicating that aberrant disulfide bonding that occurs as these mutant proteins aggregate is likely to be secondary to the assembly of aggregate structures.
